# TSC2 mediates hyperosmotic stress-induced inactivation of mTORC1

**DOI:** 10.1038/srep13828

**Published:** 2015-09-08

**Authors:** Monika Plescher, Aurelio A. Teleman, Constantinos Demetriades

**Affiliations:** 1 Division of Signal Transduction in Cancer and Metabolism, German Cancer Research Center (DKFZ), 69120, Heidelberg, Germany

## Abstract

mTOR complex 1 (mTORC1) regulates cell growth and metabolism. mTORC1 activity is regulated via integration of positive growth-promoting stimuli and negative stress stimuli. One stress cells confront in physiological and pathophysiological contexts is hyperosmotic stress. The mechanism by which hyperosmotic stress regulates mTORC1 activity is not well understood. We show here that mild hyperosmotic stress induces a rapid and reversible inactivation of mTORC1 via a mechanism involving multiple upstream signaling pathways. We find that hyperosmotic stress causes dynamic changes in TSC2 phosphorylation by upstream kinases, such as Akt, thereby recruiting TSC2 from the cytoplasm to lysosomes where it acts on Rheb, the direct activator of mTORC1. This work puts together a signaling pathway whereby hyperosmotic stress inactivates mTORC1.

The mechanistic target of rapamycin (mTOR, formerly known as mammalian target of rapamycin) senses the nutritional status of the cell and is a master regulator of growth and metabolism^1^,^2^. mTOR is a serine/threonine protein kinase that is highly conserved from yeast to mammals and belongs to the phosphatidylinositol 3-kinase (PI3K)-related kinase family (PIKK)^3^. mTOR is present in two distinct molecular complexes, mTOR complex 1 (mTORC1) and mTOR complex 2 (mTORC2), which show different subunit composition, functions, upstream regulation, and sensitivity to the allosteric mTOR inhibitor rapamycin^2^,^4^. mTORC1 integrates various upstream signals—such as growth factors, nutrients, energy levels, oxygen and stresses—to regulate protein, lipid and nucleic acid synthesis, ribosomal biogenesis, glucose uptake, glycolysis, NADPH production and autophagy, thereby controlling cellular metabolism and growth^1^,^2^. Dysregulation of mTOR is implicated in the pathophysiology of various diseases such as cancer, and metabolic disorders^1^,^2^,^5^.

One stress to which cells are exposed under both physiological and pathophysiological conditions is hyperosmotic stress. Cells within the kidney (renal inner medula), the gastrointestinal tract, the cornea, the liver, intervertebral discs and joints are exposed to substantial osmolarity fluctuations even under physiological conditions (reviewed in^6^). In addition, dietary and ambient conditions can also induce hypernatremia in sites such as the respiratory epithelium and the skin, resulting in approximate hyperosmolalities of 400 mOsm/kg^7^,^8^,^9^. Hyperosmotic stress acts as an inflammatory stimulus and is implicated in various pathophysiological conditions. Examples are inflammatory diseases of the gastrointestinal tract, liver disease, cardiovascular disease and the dry eye syndrome (reviewed in^6^). Moreover, increased plasma tonicity (in conditions of serum hyperglycemia and hypernatremia/hyperkalemia) is suggested to induce an elevation of blood pressure and the progression of diabetes, promoting insulin resistance, diabetic cataract formation, and diabetic nephropathy (renal tubular fibrosis) (reviewed in^6^). Finally, epithelial cells exposed to sublytic concentrations of bacterial pore-forming toxins can face osmotic stress^10^. Hyperosmotic stress causes cells to shrink, thereby increasing the density of all intracellular macromolecules. Since cells are sensitive to such alterations, they have developed mechanisms to quickly compensate for osmostress by regulating their volume (reviewed in^11^). If cells fail to compensate for osmostress, they trigger apoptosis and die^12^,^13^,^14^. Hyperosmotic stress has powerful and broad effects on cells, inhibiting translation, transcription and DNA replication, causing DNA and protein damage, and inducing cytoskeletal rearrangements and mitochondrial depolarization^11^,^13^.

Previous reports investigating the effect of hyperosmotic stress on mTORC1 came to differing conclusions. Most of them found that osmostress inactivates mTORC1 (as depicted by phosphorylation of its downstream substrates S6 kinase and 4E-BP1)^15^,^16^,^17^,^18^,^19^, others reported that mTORC1 activity is not significantly affected^20^ and others that mTORC1 activity increases in response to hyperosmotic conditions^21^,^22^,^23^. Therefore, how mTORC1 regulation is involved in the hyperosmotic stress response is not clear. The upstream mechanisms mediating the effects of hyperosmotic stress on mTORC1 are also partially conflicting and incompletely understood^16^,^17^,^18^,^19^,^22^,^23^,^24^,^25^,^26^. Importantly, in yeast, TOR is necessary to promote cell survival upon salt stress^27^,^28^, highlighting the significance of mTOR signaling in the osmoregulatory response in eukaryotes.

The closest upstream direct activator of mTORC1 is the small GTPase Ras homolog enriched in brain (Rheb). mTORC1 binding to active GTP-bound Rheb is essential for mTORC1 activation^29^,^30^. Rheb is localized on lysosomal and endosomal membranes and possibly other endomembranes as well^31^,^32^,^33^,^34^,^35^. Thus mTORC1 activation requires both activation of Rheb as well as mTORC1 recruitment to Rheb-containing compartments^34^. Rheb activity is controlled by the tuberous sclerosis complex (TSC) consisting of tuberous sclerosis complex 1 (TSC1, also known as hamartin), tuberous sclerosis complex 2 (TSC2, also known as tuberin) and TBC1D7^36^. TSC2 has a GTPase activating protein (GAP) domain and catalyzes conversion of active GTP-bound Rheb to its inactive GDP-bound form to inhibit mTORC1^29^,^30^.

The TSC complex appears to be a central integration point for multiple stress signals to inactivate mTORC1^37^. We previously showed that amino acid removal inactivates mTORC1 in part by recruiting TSC2 to the lysosome, where it acts on Rheb to fully release and inhibit mTORC1^38^. mTORC1-dependent, TOP mRNA translation was also shown to be inhibited via TSC2 in response to amino acid starvation^39^. Similarly, growth factor signaling to mTORC1 also involves changes in TSC2 localization^40^,^41^. Furthermore, stresses such as low energy or hypoxia were also described to signal via multiple mechanisms to the TSC complex to inhibit mTORC1^42^,^43^,^44^. Therefore, most upstream inputs that regulate mTORC1 follow diverse pathways but integrate on the TSC complex to control its activity. Whether regulation of TSC2 subcellular localization represents a general mechanism to control its function is an important question that has not been answered yet.

Here we show that mild hyperosmotic stress is capable of inactivating mTORC1 in a rapid and reversible manner. In response to activation of a calyculin-A-sensitive upstream phosphatase, osmostress leads to inhibition of Akt, thereby relocalizing TSC2 to the lysosome to act on Rheb, and therefore inhibit mTORC1. This work pieces together a pathway that leads to mTORC1 inhibition in response to hyperosmotic stress.

## Results

### Hyperosmotic stress rapidly and reversibly inactivates mTORC1 in multiple cell lines

**Figure 1:**
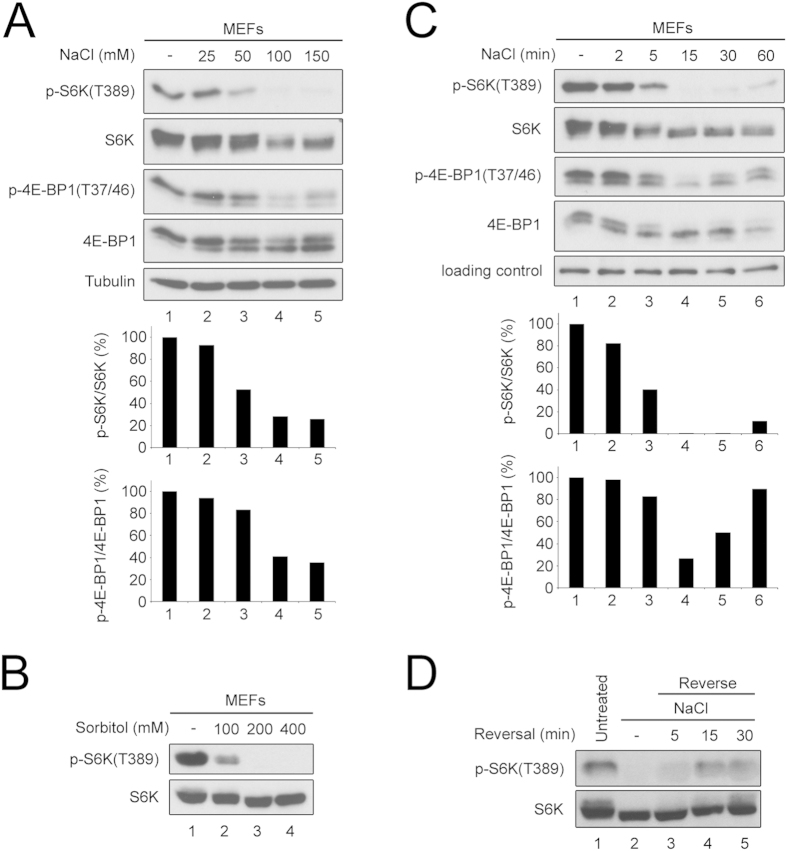
Hyperosmotic stress inactivates mTORC1 in a rapid and reversible manner. (**A**,**B**) Hyperosmotic stress inactivates mTORC1 in a dose-dependent manner. Mouse embryonic fibroblasts (MEFs) were exposed to normal or hyperosmotic culture media. Medium osmolality was increased by adding NaCl for 15 min (**A**) or sorbitol for 60 min (**B**). Lower panels show quantifications of phospho-S6K normalized to total S6K, or phospho-4E-BP1 normalized to total 4E-BP1. (**C**) Hyperosmotic stress rapidly and transiently inactivates mTORC1. Osmolality of the culture medium was increased by adding 100  mM NaCl for the indicated times. Lower panels show quantifications of phospho-S6K normalized to total S6K, or phospho-4E-BP1 normalized to total 4E-BP1. (**D**) mTORC1 inhibition by hyperosmotic stress is reversible. MEFs were treated with hyperosmotic medium (+100 mM NaCl) for 1h after which the stress was reversed by replacing with normal culture medium for the indicated time points. See also Supplementary Fig. S1.

To study the mechanism by which hyperosmotic stress (henceforth referred to as osmostress) inhibits mTORC1, we treated mouse embryonic fibroblasts (MEFs) with medium supplemented with increasing amounts of sodium chloride (NaCl), ranging from 25 mM to 150 mM. The osmolality of normal medium is roughly 330 mOsm/kg so that addition of 100 mM NaCl corresponds to a physiological increase to approximately 500 mOsm/kg^20^. In agreement with previous reports showing mTORC1 inactivation upon osmostress^15^,^16^,^17^,^18^,^19^, mTORC1 activity progressively decreases as the medium becomes more hypertonic, with 100 mM of NaCl causing nearly complete mTORC1 inactivation (Fig. 1A). This effect was due to a change in medium osmolality, and not specific for sodium chloride, since it was recapitulated by addition of the non-ionic osmolyte sorbitol (Fig. 1B). Furthermore, inactivation of mTORC1 appears to be a fairly canonical response of cells to osmostress, since we also observed it in a panel of cell lines of different origins (Supplementary Fig. S1A–C). mTORC1 inhibition occurs very rapidly in response to osmostress, reaching maximal inhibition 15 minutes after treatment and mildly recovering at later time points (Fig. 1C). Although we do not know the reason for the mild recovery in mTORC1 activity at later time points, mammalian cells are known to rapidly activate ion transport systems to compensate for altered medium osmolarity^45^. The mTORC1 inhibition is rapidly reversed upon returning cells to normal medium (Fig. 1D). For all subsequent treatments, we used osmostress conditions (+100 mM NaCl) that induce a robust effect on mTORC1, but are mild enough that cells survive it, based on reversibility of the effect.

### Osmostress causes lysosomal recruitment of TSC2

**Figure 2:**
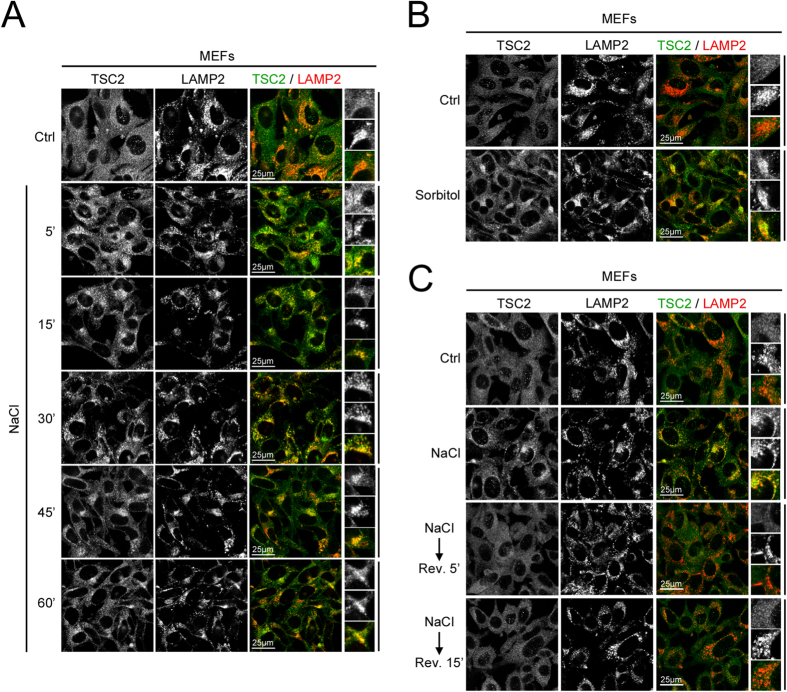
Hyperosmotic stress causes a rapid and reversible recruitment of TSC2 to lysosomes. (**A**,**B**) Hyperosmotic stress causes recruitment of TSC2 to lysosomes. Medium osmolality was increased by addition of 100 mM NaCl (**A**) or 200 mM sorbitol (**B**) for the indicated times. LAMP2 staining was used as a lysosomal marker. Representative magnified insets are shown on the right. (**C**) Lysosomal localization of TSC2 induced by hyperosmotic stress is rapidly reversible, with TSC2 returning to its original, diffusely cytosolic localization when cells are returned to medium with normal osmolality. MEFs were treated with hyperosmotic medium (+100 mM NaCl) for 1h and then the medium was replaced with normal culture medium as indicated for 5 or 15 min.

We previously reported that the mTORC1 inhibitor TSC2 rapidly accumulates on lysosomes when cells are treated with medium lacking amino acids (nutrient stress)^38^. Likewise, TSC2 relocalizes to lysosomal membranes when cells are deprived of growth factor signaling^40^,^41^. Hence, we asked whether TSC2 also relocalizes to lysosomes in response to osmostress. Indeed, treating cells with hypertonic medium caused a rapid accumulation of TSC2 to lysosomes (marked by LAMP2), regardless of whether the hypertonicity of the medium was caused by addition of NaCl (Fig. 2A) or sorbitol (Fig. 2B). Whereas TSC2 localization is uniformly cytoplasmic in MEFs treated with control medium (Fig. 2A, top row), clear lysosomal accumulations of TSC2 could be observed as early as 5 minutes after induction of osmostress (Fig. 2A, second row). Similar to the reversibility of mTORC1 activity, the lysosomal localization of TSC2 was rapidly reversed, going back to a uniform cytoplasmic distribution within 5 minutes of returning cells to isotonic medium (Fig. 2C).

### The TSC complex is required for proper inactivation of mTORC1 in response to osmostress

**Figure 3:**
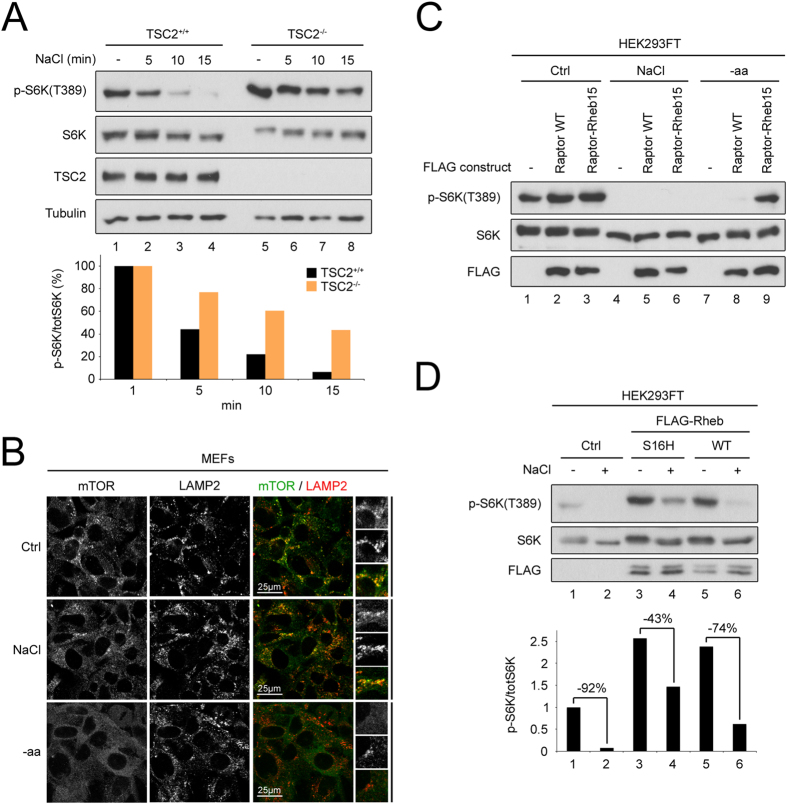
TSC2 and Rheb mediate mTORC1 inactivation upon hyperosmotic stress. (**A**) *TSC2*-null MEFs do not appropriately inactivate mTORC1 upon acute hyperosmotic stress. Control or *TSC2* knock-out MEFs were exposed to hyperosmotic media by the addition of 100 mM NaCl for the indicated times prior to lysis and immunoblotting. (**B**) Hyperosmotic stress does not affect mTOR localization. MEFs were treated with hyperosmotic medium (+100 mM NaCl) or medium lacking amino acids (-aa) for 1 h and mTOR localization was analyzed by immunofluorescence and confocal microscopy. LAMP2 staining was used as a lysosomal marker. Whereas mTOR localization becomes diffusely cytoplasmic upon removal of amino acids, it remains lysosomally concentrated upon hyperosmotic stress. Representative magnified insets are shown on the right. (**C**) Hyperosmotic stress inhibits mTORC1 independently of its localization. mTORC1 in HEK293FT cells was targeted to Rheb-containing endomembranes by overexpression of chimeric Raptor-Rheb15, as previously described^34^. mTORC1 activity was analyzed for non-treated cells (Ctrl), cells treated for 15 min with hyperosmotic medium (+100 mM NaCl) or 1 h with medium lacking amino acids (-aa). (**D**) Rheb^GTP^ overexpression partially rescues mTORC1 inactivation upon hyperosmotic stress. Wild-type (WT) or an active, GTP-locked, TSC2-insensitive Rheb mutant (S16H) were expressed in HEK293FT cells and mTORC1 activity was analyzed in cells treated with normal medium (−) or hyperosmotic medium (+) for 15 min (top). Quantification and the percent drop of normalized S6K phosphorylation is also shown (bottom). See also Supplementary Fig. S2.

To test the requirement for TSC2 in the response of mTORC1 to osmostress, we treated control and *TSC2* knock-out MEFs with hypertonic medium. Whereas control cells rapidly inactivate mTORC1 within minutes of osmostress induction, *TSC2*-null MEFs retain significant amounts of mTORC1 activity (Fig. 3A). More extended treatments with hypertonic medium led to complete mTORC1 inactivation also in *TSC2*-null MEFs (Supplementary Fig. S2A), indicating that TSC2 is required for the initial inactivation of mTORC1 in response to osmostress, whereas a TSC2-independent mechanism inactivates mTORC1 at later time points. In this report, we focus on the initial mTORC1 inactivation that occurs within the first 5–15 minutes of osmostress that is TSC2-dependent. As expected, delayed inactivation of mTORC1 in response to osmostress was also observed in MEFs lacking the TSC2 binding partner TSC1 (Supplementary Fig. S2B).

mTORC1 activity is regulated via a combination of two factors—its subcellular localization and the state of activation of its binding partner Rheb^34^. Hence, we asked whether osmostress causes displacement of mTORC1 away from lysosomes, but this was not the case. Whereas mTORC1 becomes cytoplasmic in cells deprived of amino acids (“-aa”, Fig. 3B), it was still lysosomally concentrated in cells treated with hypertonic medium (“NaCl”, Fig. 3B). This suggests osmostress does not regulate mTORC1 via its subcellular localization, but rather via the state of Rheb activation. Indeed, forcing mTORC1 to localize to Rheb-containing endomembranes, by expressing a Raptor-Rheb chimera does not prevent mTORC1 inactivation in response to osmostress (Fig. 3C). As a control, the Raptor-Rheb chimera rescues mTORC1 inactivation upon amino acid starvation (“-aa” in Fig. 3C), as previously reported^34^. Consistent with osmostress regulating Rheb activation rather than mTORC1 localization, expression of a constitutively active Rheb that cannot be acted upon by TSC2^46^ partially rescues mTORC1 activity in response to osmostress (lanes 3–4, Fig. 3D), whereas expression of wild-type Rheb is less potent (lanes 5–6, Fig. 3D). Consistent with the existence of a TSC2/Rheb-independent mechanism that inactivates mTORC1 at later time points, overexpression of constitutively active Rheb was not sufficient to prevent mTORC1 inhibition by prolonged osmostress (Supplementary Fig. S2C).

### Osmostress regulates multiple signaling pathways impinging upon TSC2

**Figure 4:**
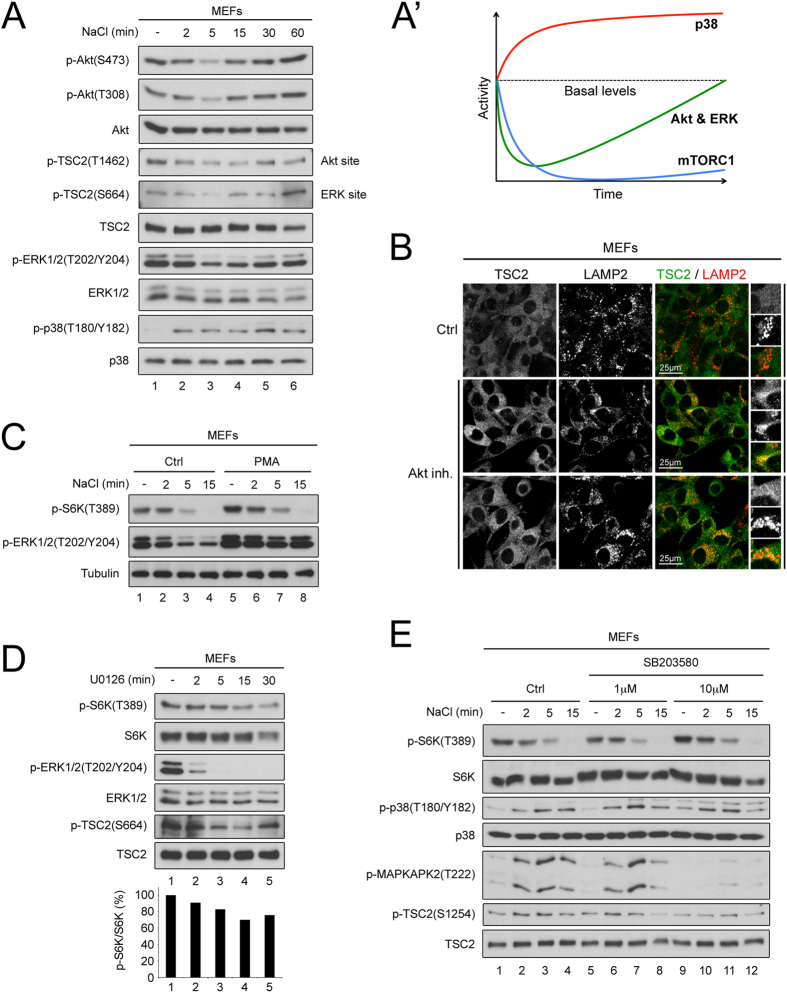
Hyperosmotic stress regulates multiple signaling pathways upstream of TSC2 to cause its lysosomal localization. (**A**-**A**′) Hyperosmotic stress rapidly regulates multiple signaling pathways impinging upon TSC2. Osmolality of the culture medium was increased by adding 100 mM NaCl for the indicated times. Schematic representation of the kinetic changes in the signaling pathways is shown in (**A**′). (**B**) Akt inhibition is sufficient to cause mild TSC2 lysosomal localization. MEFs were treated with 10 μM Akt inhibitor VIII for 15 min and TSC2 localization was analyzed by immunofluorescence and confocal microscopy. LAMP2 staining was used as a lysosomal marker. Representative magnified insets are shown on the right. (**C**–**E**) The ERK and p38MAPK pathways are not sufficient to mediate hyperosmotic stress-induced inhibition of mTORC1. (**C**) Sustained ERK1/2 activity does not prevent mTORC1 inhibition in response to hyperosmotic stress. MEFs were pretreated with 1 μg/ml phorbol 12-myristate 13-acetate (PMA) for 40 min and then exposed to hyperosmotic stress (+100 mM NaCl) for the indicated times. (**D**) Inhibition of MEK/ERK kinases does not phenocopy the mTORC1 inhibition observed in response to hyperosmotic stress. MEFs were treated with 25 μM MEK inhibitor U0126 for the indicated times prior to lysis and immunoblotting. (**E**) Inhibition of p38MAPK does not prevent mTORC1 inactivation in response to hyperosmotic stress. MEFs were pretreated with the indicated concentrations of p38 inhibitor SB203580 and then exposed to hyperosmotic stress for the indicated times. See also Supplementary Fig. S3.

The TSC1/2 complex is a central integration point for the regulation of mTORC1 in response to upstream stimuli^37^,^38^. Phosphorylation of the TSC1/2 complex is regulated by multiple signaling pathways including Akt, Erk, AMPK, RSK, GSK3β and p38/MAPKAPK2^37^,^47^, many of which have been previously reported to be affected by osmostress^11^,^26^. We analyzed which of these pathways are modulated in our experimental setup. Treatment of MEFs with hypertonic medium leads to a transient decrease in phosphorylation of Akt on both the PDK1 (Thr308) and mTORC2 (Ser473) sites (Fig. 4A and Supplementary Fig. S1A–C), in agreement with two previous reports^16^,^24^. The drop in Akt phosphorylation leads to a decrease in its activity, as reflected by the dephosphorylation of its canonical target GSK3β (Supplementary Fig. S3A) and a corresponding drop in phosphorylation of TSC2 on the Akt site (Thr1462) (Fig. 4A, and Supplementary Fig. S1B-C and S3A). Osmostress also causes a transient drop in Erk phosphorylation (Fig. 4A and Supplementary Fig. S3B) and in phosphorylation of TSC2 on Ser664, the Erk site^48^,^49^ (Fig. 4A). In contrast with the transient regulation of the other kinases, p38 phosphorylation increases steadily in response to osmostress (Fig. 4A). The phosphorylation of several other kinases tested was unaffected by osmostress (Supplementary Fig. S3B-B′). In sum, osmostress modulates activity of various signaling pathways that impinge on TSC2 (summarized in Fig. 4A′).

### Lysosomal recruitment of TSC2 is mainly mediated via modulation of Akt signaling

Since osmostress modulates the activity of multiple pathways affecting TSC2, we aimed to dissect which ones are principally responsible for the lysosomal recruitment of TSC2 and the TSC2-mediated inhibition of mTORC1. Pharmacological inhibition of Akt caused both inhibition of mTORC1 (Supplementary Fig. S3C) and a mild but significant recruitment of TSC2 to lysosomes (Fig. 4B), as previously reported (in *PTEN*-null MEFs^41^), indicating that inhibition of Akt contributes towards mTORC1 inactivation and lysosomal recruitment of TSC2 in response to osmostress. In contrast, inhibition of Erk and activation of p38 signaling in response to osmostress appear to play less of a role in the response of mTORC1. Pretreatment of MEFs with phorbol 12-myristate 13-acetate (PMA) prevented the inhibition of Erk in response to osmostress (p-ERK1/2, Fig. 4C), but did not rescue the inactivation of mTORC1 (p-S6K, Fig. 4C). Furthermore, use of a MEK inhibitor (U0126) to inactivate Erk only had a weak effect on mTORC1 activity, and only at later time points (Fig. 4D). Likewise, pharmacological inhibition of p38 with 10 μM SB203580 prevented the phosphorylation of its downstream target MAPKAPK2 in response to osmostress (Fig. 4E) but did not prevent the inactivation of mTORC1 (p-S6K, Fig. 4E), in agreement with a previous report^21^. Both Erk and p38 inhibition resulted in the expected changes in phosphorylation of TSC2 on the respective sites (Ser664 and Ser1254) (Fig. 4D,E)^48^,^49^,^50^, suggesting that these phosphorylations are not sufficient to lead to TSC2 activation upon osmostress. In sum, the inhibition of mTORC1 and recruitment of TSC2 to lysosomes in response to osmostress is mainly mediated via Akt.

### Osmostress inhibits Akt and S6K via activation of a Calyculin-A-sensitive phosphatase

**Figure 5:**
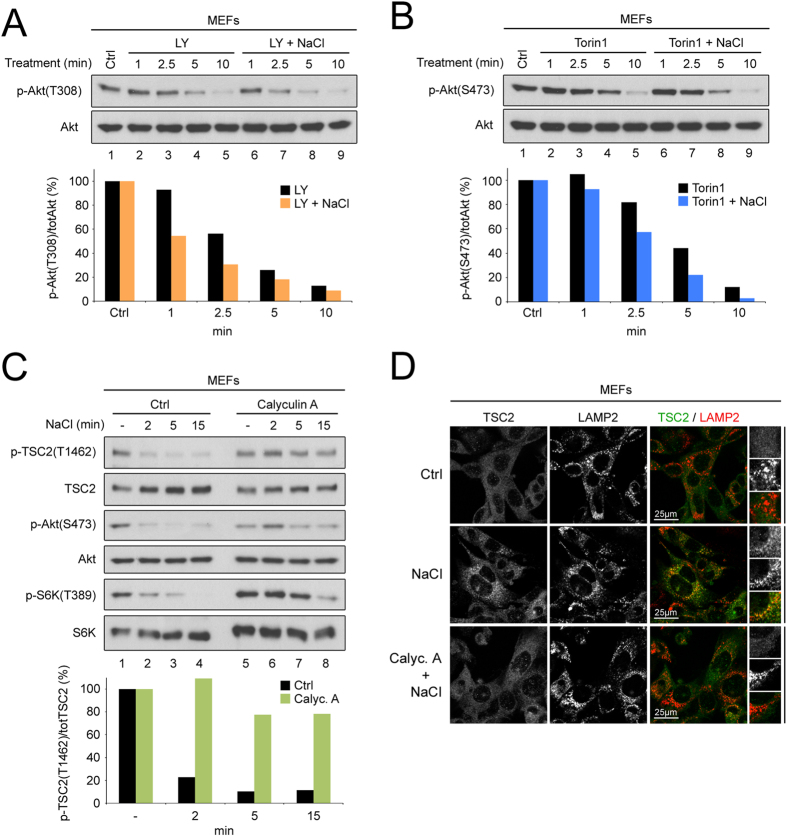
Hyperosmotic stress activates a Calyculin-A-sensitive phosphatase to regulate Akt and S6K. (**A**,**B**) Phosphatase activity on Akt is increased upon hyperosmotic stress. Time courses of treatment with 50 μM LY294002 (**A**) or 250 nM Torin1 (**B**) were performed to inactivate PDK1 or mTORC2 respectively, and observe the rate of dephosphorylation of the respective sites on Akt, both in the absence of hyperosmotic stress or when NaCl is added simultaneously with the drug (+100 mM NaCl). In the presence of hyperosmotic stress, both the PDK1 (**A**) and the mTORC2 (**B**) sites on Akt become dephosphorylated more quickly than in control conditions. Quantifications of phospho-Akt normalized to total Akt are shown in the lower panels. (**C**) Inhibition of a calyculin-A-sensitive phosphatase prevents TSC2 and Akt dephosphorylation and mTORC1 inactivation upon hyperosmotic stress. MEFs were pretreated with DMSO (“Ctrl”) or 15 nM Calyculin A for 30 min and then exposed to hyperosmotic medium (+100 mM NaCl) for the indicated times. Lower panel shows quantification of phospho-TSC2 normalized to total TSC2 levels. (**D**) Inhibition of a calyculin-A-sensitive phosphatase rescues the osmostress-induced lysosomal accumulation of TSC2. MEFs were left untreated (“Ctrl”) or treated with 100 mM NaCl for 15 min following pretreatment with DMSO (“NaCl”) or 15 nM Calyculin A for 5 min (“Calyc. A + NaCl”). TSC2 localization was analyzed by immunofluorescence and confocal microscopy. LAMP2 staining was used as a lysosomal marker. Representative magnified insets are shown on the right. See also Supplementary Fig. S4.

Since the main contribution towards mTORC1 regulation in the response to osmostress is via Akt, we aimed to delineate the mechanism of Akt inactivation. Although previous reports have come to differing conclusions regarding the effect of osmostress on Akt activity^16^,^22^,^24^,^25^, our data are in line with previous work, which shows that Akt is inhibited by a calyculin-A-sensitive phosphatase^16^,^24^. We performed a time course to analyze the rate of dephosphorylation of Akt on the PDK1 and mTORC2 sites. Treatment of MEFs with LY294002 to inhibit PI3K and hence PDK1 leads to dephosphorylation of Akt on the PDK1 site (Thr308) within 5 minutes (Fig. 5A lanes 1–5). When PDK1 is inhibited in the presence of osmostress, the rate of dephosphorylation on this site is increased (compare lanes 7 to 3 and 8 to 4, Fig. 5A), indicating elevated phosphatase activity. Likewise, inhibition of mTORC2 with Torin1 leads to dephosphorylation of Akt on the mTORC2 site (Ser473) (Fig. 5B lanes 1–5), which is accelerated in the presence of osmostress (Fig. 5B lanes 6–9, compare 5 and 10  minute time points). Pretreatment of MEFs with calyculin A, an inhibitor of PP2A and PP1 phosphatases, prevented dephosphorylation of Akt, the Akt phosphosite on TSC2 (Thr1462) and impaired mTORC1 inactivation in response to osmostress (Fig. 5C). In line with the effect of calyculin A on TSC2 phosphorylation by Akt, pretreatment with this drug also abolished the lysosomal relocalization of TSC2 in response to osmostress (Fig. 5D).

**Figure 6:**
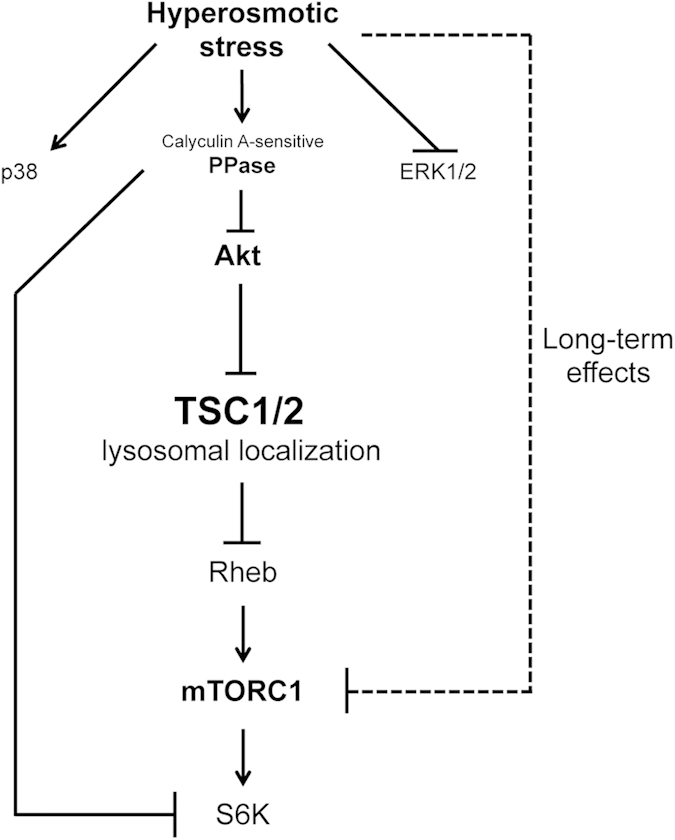
Proposed model for hyperosmotic stress-induced inhibition of mTORC1. Hyperosmotic stress rapidly inactivates Akt via activation of a Calyculin-A-sensitive phosphatase and regulates other signaling pathways (ERK1/2, p38MAPK) independently of this phosphatase. Subsequently, TSC2 phosphorylation changes and its lysosomal localization increases, thereby inhibiting Rheb and mTORC1, as indicated by phosphorylation of its direct substrate S6K. In addition, the Calyculin-A-sensitive phosphatase acts on S6K itself to inactivate it. At later time points, additional TSC2/Rheb-independent pathways also contribute to mTORC1 inhibition.

Previous reports showed that S6K dephosphorylation is also mediated by a calyculin-A-sensitive phosphatase, such as PP2A^15^,^51^. We therefore tested if a calyculin-A-sensitive phosphatase is also acting on S6K in our experimental setup. Indeed, osmostress accelerated (Supplementary Fig. 4A) and calyculin A pretreatment prevented (Supplementary Fig. 4B) the dephosphorylation of S6K in response to the mTOR inhibitor Torin1. In sum, osmostress activates a calyculin-A-sensitive phosphatase that dephosphorylates Akt, thereby leading to TSC2 activation and accumulation to lysosomes, and in parallel acts on the mTORC1 substrate S6K to cause its dephosphorylation (Fig. 6).

## Discussion

Previous reports showed that osmostress inhibits mTORC1^15^,^16^,^17^,^18^,^19^, however the mechanism of mTORC1 inactivation was unclear. We show here that osmostress affects TSC2 phosphorylation and subcellular localization, thereby inhibiting mTORC1 via Rheb. Combining our data with data from the literature yields a signaling pathway whereby osmostress activates a calyculin-A-sensitive phosphatase, which dephosphorylates Akt on both the PDK1 and mTORC2 sites (Fig. 6). This leads to inactivation of Akt, loss of TSC2 phosphorylation on the Akt site (Thr1462), and accumulation on lysosomes where it inactivates Rheb and thereby mTORC1 (Fig. 6). Effects of osmostress on the activity of other kinases, such as p38 and Erk, and the phosphorylation of the respective sites on TSC2 might also contribute to its activation, although they were not sufficient to affect mTORC1 activity, when activated or inhibited singly. In agreement with previous reports^15^,^51^, the calyculin-A-sensitive phosphatase also acts on the mTORC1 substrate S6K to dephosphorylate it, thereby providing a possible explanation for the partial TSC2/Rheb-independent drop in S6K phosphorylation upon osmostress.

We previously reported that another stress—amino acid starvation—also causes TSC2 to accumulate on lysosomes^38^. There are some similarities and some differences between osmostress and amino acid removal that are worth noting. In both cases, TSC2 rapidly relocalizes to lysosomes and acts on Rheb. However, in the case of amino acid withdrawal, this leads to displacement of mTORC1 away from lysosomes, whereas in the case of osmostress this does not occur. There are two tethering activities keeping mTORC1 on the lysosome—the Rag GTPases^18^,^38^,^52^ and Rheb^38^. Amino acid removal severs both tethering activities; the Rag GTPases release mTORC1 as a result of GAP activity by the GATOR1 complex^53^, and Rheb releases mTORC1 as a result of inhibition by TSC2. In contrast, in response to osmostress, the Rag GTPases continue to tether mTORC1 on the lysosome, since they are inactivated specifically by amino acid withdrawal^18^.

Unlike the response of mTORC1 to other stresses, such as removal of growth factor signaling, mTORC1 inactivation in response to osmostress is extremely rapid, taking only a few minutes. Presumably, this is because osmostress is very noxious to the cell^12^,^13^,^14^,^54^. This initial, rapid inactivation of mTORC1 to osmostress appears to be partly TSC-dependent (Fig. 3A and Supplementary Fig. S2B), and was the focus of this present study. Two pieces of data suggest, however, that additional TSC2- and Rheb-independent mechanisms exist to inactivate mTORC1 and its downstream substrates both during the early response to osmostress (5–10 min after treatment) and on a longer timescale (60 min after treatment, Fig. 6). Firstly, although mTORC1 inactivation in response to osmostress is clearly impaired in *TSC2*-null MEFs, they nonetheless do show some mTORC1 inactivation (Supplementary Fig. S2A and 55). Secondly, expression of active, GTP-locked Rheb does not fully rescue mTORC1 inactivation in response to osmostress at early time points (Fig. 3D) and is not sufficient to prevent mTORC1 inhibition at late time points (60 minutes in Supplementary Fig. S2C). This is unlikely to be due to technical issues, since in our hands the activated Rheb fully rescues mTORC1 inactivation upon 60 minutes of amino acid withdrawal (Supplementary Fig. S2C), in agreement with previous reports^29^,^30^. This suggests osmostress likely activates multiple mechanisms that act in parallel to inhibit mTORC1 and its downstream effectors such as S6K. Indeed, osmostress activates a calyculin-A-sensitive phosphatase acting directly on S6K (Supplementary Fig. 4 and^15^,^51^). Furthermore, at later time points osmostress leads to phosphorylation of Raptor by MARK4^19^. It is possible that osmostress also causes additional effects such as dissociation of the mTORC1 complex, similar to what is seen in response to energetic stress^56^. In agreement with this, a previous report found that Rheb overexpression has a mild effect if cells are treated with high sorbitol concentrations (600 mM) for longer time points (30 min)^29^. In sum, several mechanisms ensure robust inactivation of mTORC1 and S6K in response to osmostress, with the early response being largely TSC2-dependent.

## Materials and methods

### Cell Culture

Immortalized mouse embryonic fibroblasts (MEFs), and embryonic kidney HEK293FT (Invitrogen), were cultured in high-glucose Dulbecco’s modified Eagle’s medium (DMEM) (#11965–092, Gibco), supplemented with 10% FBS (Biochrom). HeLa and MCF-7 cells were cultured in high-glucose DMEM containing 10% FBS (PAA). MCF-7 cells were also supplemented with 1× non-essential amino acids (Gibco). All media were supplemented with 1× Penicillin-Streptomycin (Gibco). *TSC1*^*−/−*^, *TSC2*^*+/+*^*p53*^*−/−*^, and *TSC2*^*−/−*^*p53*^*−/−*^ MEFs were a kind gift by David Kwiatkowski and Michael Hall and were described previously^57^,^58^.

The identity of HEK293FT, HeLa, and MCF-7 cells was verified using the Multiplex human Cell line Authentication service (MCA, Multiplexion GmbH), which uses an SNP-profiling approach and was performed as described at www.multiplexion.de. The HEK293FT, HeLa, MCF-7, TSC2^+/+^p53^−/−^ and TSC2^−/−^p53^−/−^ MEF cell lines were verified to be *Mycoplasma*-free and free of contamination with cells of other species, according to the Multiplex cell Contamination Test Report (Multiplexion GmbH), as described at www.multiplexion.de.

### Cell treatments and media composition

Hyperosmotic stress conditions were applied by addition of NaCl or sorbitol to the culture media. The concentration of NaCl in serum-free normal culture media (high-glucose DMEM, Gibco) is 110.35 mM and the overall osmolality is 320–360 mOsm/kg, according to the manufacturer’s specifications. An increase of the NaCl concentration by 100 mM or addition of 200 mM sorbitol to full, serum-containing media raises osmolality to ≤500 mOsm/kg^20^. For reversal of hyperosmotic stress, the hypertonic media were removed and replaced by normal culture media.

Amino acid starvation was carried out as previously described^38^. In brief, the culture media were replaced with treatment media containing or lacking only all amino acids. Treatment media were supplemented with 10% dialyzed FBS. The starvation media were formulated according to the Gibco recipe for high-glucose DMEM, omitting the amino acids.

Pharmacological treatments using Akt inhibitor VIII (#124018, Calbiochem), MEK1/2 inhibitor U0126 (#9903, Cell Signaling Technology), PKC activator phorbol 12-myristate 13-acetate (PMA) (#P8139, Sigma), p38MAPK inhibitor SB203580 (#AG-CR1–0030-M001, AdipoGen), PI3K inhibitor LY294002 (#9901, Cell Signaling Technology), mTOR inhibitor Torin1 (#Cay10997, Cayman Chemical), and phosphatase inhibitor Calyculin A (sc-24000, Santa Cruz Biotechnology), were performed as described in the figure legends.

### Antibodies

Antibodies against phospho-S6K(T389) (#9205), S6K (#9202), phospho-4E-BP1(T37/46) (#9459), 4E-BP1 (#9452), phospho-Akt(S473) (#9271), phospho-Akt(T308) (#9275), Akt (#9272), phospho-TSC2(T1462) (#3611), phospho-TSC2(S1387) (#5584), phospho-TSC2(S1254) (#3616), TSC2 (#4308), mTOR (#2983), phospho-GSK3β(Ser9) (#5558), phospho-p38(T180/Y182) (#9216), p38 (#9212), phospho-MAPKAPK2(T222) (#3316), phospho-ERK1/2(T202/Y204) (#4370), ERK1/2 (#4695), phospho-AMPKβ1(S108) (#4181), phospho-p90RSK(S380) (#9341), phospho-Erk5(T218/Y220) (#3371), phospho-CaMKII(T286) (#3361), phospho-MST1(T183)/MST2(T180) (#3681), and phospho-YAP(S127) (#4911) proteins were purchased from Cell Signaling Technology. An antibody against phospho-TSC2(S664) was purchased from Abcam (ab133465). The mouse LAMP2 (ABL-93) antibody was obtained from Developmental Studies Hybridoma Bank. A monoclonal antibody recognizing human and mouse α-tubulin (#T9026) and an anti-FLAG (M2) (#F1804) antibody were purchased from Sigma. All antibodies were used in 1:1000 dilution for western blotting, except for the phospho-ERK, total ERK, TSC2, and p38 antibodies that were used in 1:2000.

### Cell lysis and Western blotting

For SDS-PAGE and immunoblotting experiments, cells were lysed in-well with ice-cold Triton lysis buffer (50 mM Tris pH 7.5, 1% Triton X-100, 150 mM NaCl, 50 mM NaF, 2 mM Na-vanadate, 0.011 gr/ml beta-glycerophosphate, 1× PhosSTOP phosphatase inhibitors and 1× Complete protease inhibitors) for 10 minutes on ice. Samples were clarified by centrifugation (15 min, 14,000 rpm, 4 °C), and SDS loading buffer was added to the supernatant before boiling. The samples were analyzed by 1D gel electrophoresis and phospho- and total protein levels were detected using the appropriate antibodies. Quantification of immunoblots was performed using the LICOR Fc detection system and the ImageStudio software (most panels) or by densitometry of films with ImageJ (p-4E-BP1 panels in Figs 1A,C, 4D and 5C).

### Plasmid constructs

For the pcDNA3-FLAG-Rheb WT expression vector, full-length *Rheb* was amplified from human cDNA with oligos containing the appropriate restriction enzyme overhangs and cloned in the EcoRI/NotI sites, in frame with the FLAG tag sequence in pcDNA3-FLAG. The S16H point mutant was created by site-directed mutagenesis. A similar vector expressing FLAG-tagged firefly Luciferase was used as a negative control (pcDNA3-FLAG-Luc). The *Luciferase* gene was amplified from a pGL3 vector (Promega) and cloned in the EcoRI/NotI sites, in frame with the FLAG tag sequence in pcDNA3-FLAG. The integrity of all constructs and the presence of point mutations was verified by sequencing.

The pLJM1-FLAG-Raptor WT and pLJM1-FLAG-Raptor-Rheb15 were a gift from David Sabatini (Addgene plasmids #26633 and 26634) and have been described previously^34^.

### Plasmid transfections

Plasmid DNA transfections in HEK293FT cells were performed using Effectene (QIAGEN), according to manufacturer’s instructions.

### Cell Imaging/Immunofluorescence

Immunofluorescence experiments were performed as previously described^38^. In brief, cells were seeded on fibronectin-coated glass coverslips and treated as indicated in each experiment. Following treatments, cells were fixed for 10 min at room temperature with 4% PFA in PBS. Samples were washed/permeabilized twice with PBT solution (1× PBS, 0.1% Tween-20) for 10 min, and blocked with BBT solution (1× PBS, 0.1% Tween-20, 0.1% BSA) for 45 min. Stainings were performed with the indicated primary antibodies diluted in BBT (1:100–1:200) for 2 h, following 1 h incubation with appropriate highly cross-adsorbed secondary fluorescent antibodies. Nuclei were stained with DAPI and the coverslips were mounted on slides using a glycerol-based mounting medium (80% glycerol, 1× PBS, 0,4% propyl gallate). Images from single channel captures are shown in grayscale. For the merged images, FITC is shown in green, and TRITC in red. The images were captured using a 40× objective lens and 3× zoom on an SP8 Leica confocal microscope. All cell images within each panel were acquired and displayed using the same settings.

## Additional Information

**How to cite this article**: Plescher, M. *et al.* TSC2 mediates hyperosmotic stress-induced inactivation of mTORC1. *Sci. Rep.* **5**, 13828; doi: 10.1038/srep13828 (2015).

## Supplementary Material

Supplementary Information
