# Supplementary material for: TSC2 mediates hyperosmotic stress-induced inactivation of mTORC1
Source: Sci Rep. 2015 Sep 8;5:13828. doi: 10.1038/srep13828 (PMC4642562; doi:10.1038/srep13828)

Supplementary Information

TSC2 mediates hyperosmotic stress-induced inactivation of mTORC1

Monika Plescher, Aurelio A. Teleman and Constantinos Demetriades

Contents

1. Supplementary Figure Legends .... p. 1

2. Supplementary Figures .... p. 3

1. Supplementary Figure Legends

Supplementary Fig. S1: Related to Fig. 1

(A-C) Hyperosmotic stress inhibits mTORC1 and Akt in various different cell lines: HEK293FT (A), HeLa (B) and MCF-7 (C) cells. Osmolality of the culture medium was increased by adding 100 mM NaCl for the indicated times.

Supplementary Fig. S2: Related to Fig. 3.

(A) mTORC1 is still inactivated in *TSC2*-null MEFs upon prolonged osmostress conditions. Wild-type and *TSC2*-null MEFs were treated with NaCl (+100 mM) for short (10 min) or longer time periods (60 min) and mTORC1 activity was analyzed by immunoblotting.

(B) *TSC1*-null MEFs show impaired mTORC1 inactivation in response to acute hyperosmotic stress. *TSC1* knockout MEFs were exposed to hyperosmotic medium (+100 mM NaCl) for the indicated times prior to lysis and immunoblotting.

(C) A Rheb-independent pathway also contributes to mTORC1 inhibition at later time points of osmostress. Active Rheb (S16H) was overexpressed in HEK293FT and cells were left untreated (Ctrl) or treated with NaCl or amino acid-depleted media (-aa) for the indicated times. mTORC1 activity was analyzed by immunoblotting.

Supplementary Fig. S3: Related to Fig. 4.

(A) Hyperosmotic stress inhibits Akt phosphorylation and activity. MEFs were treated with hyperosmotic medium (+100 mM NaCl) for the indicated times and Akt phosphorylation as well as phosphorylation of its downstream targets GSK3β and TSC2 were analyzed by immunoblotting.

(B-B') Regulation of various signaling pathways by hyperosmotic stress in MEFs. Osmolality of the culture medium was increased by adding 100 mM

NaCl for the indicated times. ERK phosphorylation changes in response to osmostress (B), but other kinases remain unaffected (B-B').

(C) mTORC1 is efficiently inhibited by treating MEFs with 10 μM Akt inhibitor VIII. MEFs were treated with the indicated amounts of Akt inhibitor VIII for 30 minutes, and mTORC1 activity was analyzed by immunoblotting.

Supplementary Fig. S4: Related to Fig. 5.

(A-B) Hyperosmotic stress activates a calyculin-A-sensitive phosphatase that also acts on S6K to regulate its phosphorylation. (A) mTORC1 was inhibited using Torin1 in the presence or absence of 100 mM NaCl for the indicated times. S6K phosphorylation was analyzed by immunoblotting. Note that when NaCl is added along with Torin1, S6K is dephosphorylated faster. (B) mTORC1 was inhibited by Torin1 in the presence or absence of Calyculin A. Cells were pre-treated with Calyculin A (15 nM) for 15 min before Torin1 addition and then Torin1 (250 nM) was added for the indicated times. Note that S6K phosphorylation is largely retained in the presence of Calyculin A.

2. Supplementary Figures

(on next page)

Demetriades et al. Supplementary Fig. S1

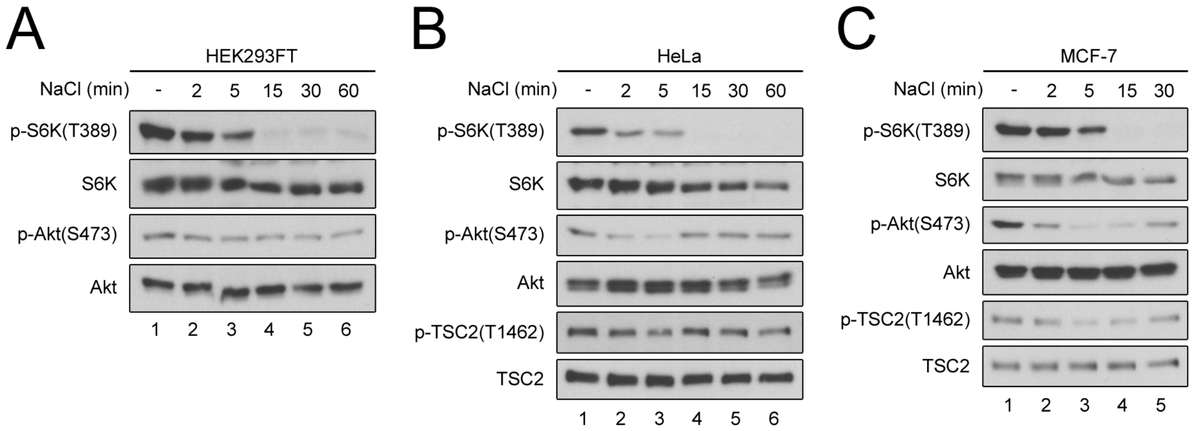

Demetriades et al. Supplementary Fig. S2

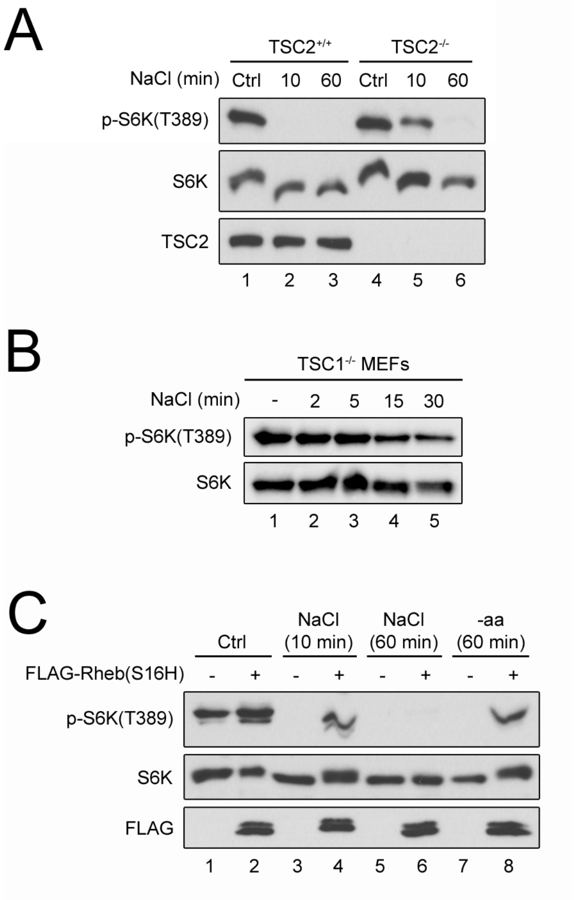

Demetriades et al. Supplementary Fig. S3

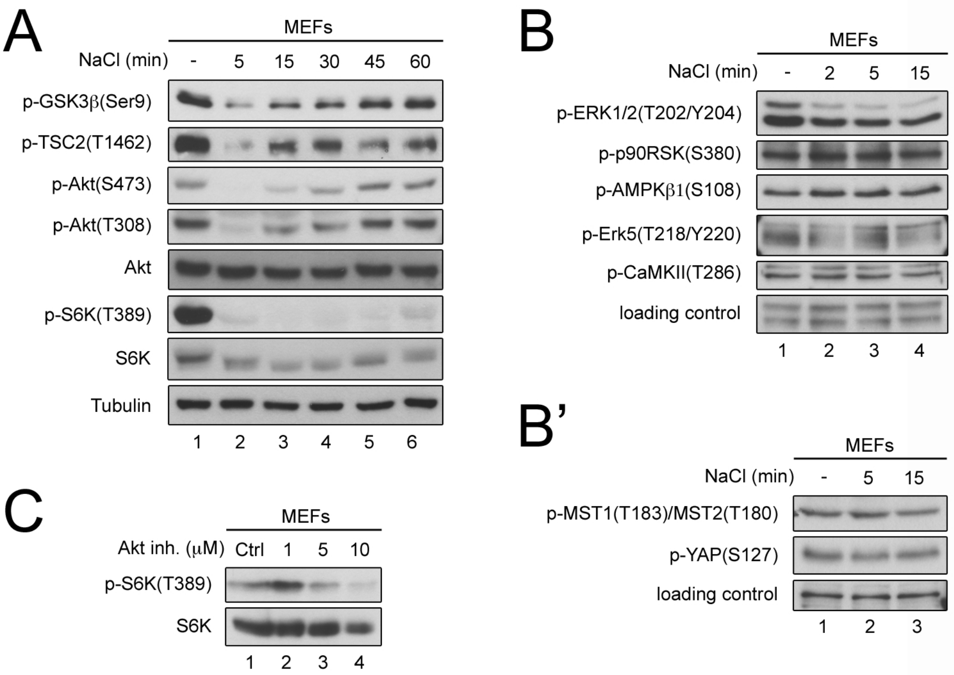

Demetriades et al. Supplementary Fig. S4

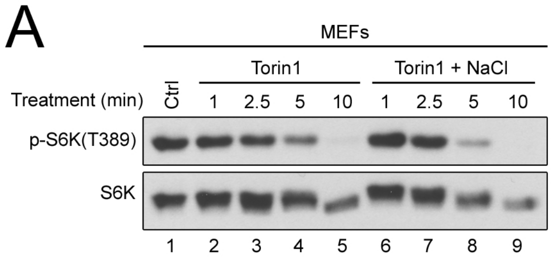

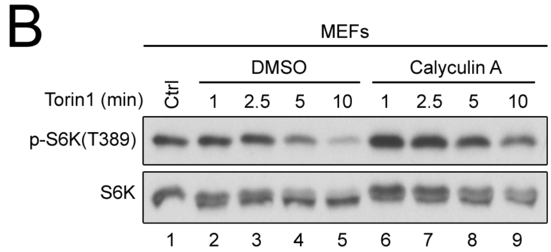

Supplement: Supplementary Information [file srep13828-s1.pdf]
